# Comparative genomic analysis reveals genetic features related to the virulence of *Bacillus cereus* FORC_013

**DOI:** 10.1186/s13099-017-0175-z

**Published:** 2017-05-15

**Authors:** Hyun-Jin Koo, Sojin Ahn, Han Young Chung, Suyeon Kim, Kwondo Kim, Sangryeol Ryu, Ju-Hoon Lee, Sang Ho Choi, Heebal Kim

**Affiliations:** 1 Department of Agricultural Biotechnology and Research Institute of Agriculture and Life Sciences, Seoul National University, Seoul, Republic of Korea; 2 Interdisciplinary Program in Bioinformatics, Seoul National University, Seoul, Republic of Korea; 3 Food-Borne Pathogen Omics Research Center (FORC), Seoul National University, Seoul, Republic of Korea; 4 Department of Agricultural Biotechnology, Center for Food Safety and Toxicology, Seoul National University, Seoul, Republic of Korea; 5 Department of Food Science and Biotechnology, Institute of Life Science and Resources, Kyung Hee University, Yongin, Republic of Korea

**Keywords:** *Bacillus cereus*, Virulence, Comparative genomics, Positive selection, Unique genes

## Abstract

**Background:**

*Bacillus cereus* is well known as a gastrointestinal pathogen that causes food-borne illness. In the present study, we sequenced the complete genome of *B. cereus* FORC_013 isolated from fried eel in South Korea. To extend our understanding of the genomic characteristics of FORC_013, we conducted a comparative analysis with the published genomes of other *B. cereus* strains.

**Results:**

We fully assembled the single circular chromosome (5,418,913 bp) and one plasmid (259,749 bp); 5511 open reading frames (ORFs) and 283 ORFs were predicted for the chromosome and plasmid, respectively. Moreover, we detected that the enterotoxin (NHE, HBL, CytK) induces food-borne illness with diarrheal symptom, and that the pleiotropic regulator, along with other virulence factors, plays a role in surviving and biofilm formation. Through comparative analysis using the complete genome sequence of *B. cereus* FORC_013, we identified both positively selected genes related to virulence regulation and 224 strain-specific genes of FORC_013.

**Conclusions:**

Through genome analysis of *B. cereus* FORC_013, we identified multiple virulence factors that may contribute to pathogenicity. These results will provide insight into further studies regarding *B. cereus* pathogenesis mechanism at the genomic level.

**Electronic supplementary material:**

The online version of this article (doi:10.1186/s13099-017-0175-z) contains supplementary material, which is available to authorized users.

## Background

For several decades, food-borne illnesses caused by microorganisms have attracted political and media attention around the world [1], because outbreaks of these diseases have a strong association with public health problems such as hospitalizations and even deaths [2, 3]. Preventing these illnesses is challenging, involving a complicated process rather than simple conventional methods [4]. Therefore, it is essential that we elucidate the phenotypic and genotypic features of agents that cause outbreaks. Among a variety of organisms that can cause food-borne illness, *Bacillus,* which is characterized by several features—a gram-positive, rod-shaped, motile, spore-forming and aerobic-to-facultative—has been considered an important opportunistic pathogen and may be categorized as (1) gastrointestinal pathogens inducing emetic and diarrheal symptoms and (2) systemic and local infections associated with the respiratory tracts of immunologically compromised patients and neonates [5, 6]. There were food poisoning outbreaks from consuming food including meat, soups, vegetable dishes, dairy products, and seafood that were contaminated with *Bacillus cereus* [7, 8]. Especially, *B. cereus* spore, which survives within the small intestine of the host, has a connection with food-borne illness inducing diarrheal symptom [9, 10]. A recent study reported that enterotoxins might cause diarrheal food-borne illness, including hemolysin BL (HBL), non-hemolytic enterotoxin (NHE) and cytotoxin K (CytK) [7, 11]. Although many research efforts have focused on the food-borne diseases caused by *B. cereus*, little genomic research of this species has been conducted into the mechanism of toxicity leading to food poisoning.

In the present study, we sequenced the complete genome of *B. cereus* FORC_013, to better understand its pathogenesis at the genomic level, which was isolated from fried eel in South Korea. Using this sequence data, we assembled the complete genome of *B. cereus* FORC_013 and determined its genomic characteristics. Then, we conducted a comparative genome analysis of the FORC_013 with the complete sequences of 29 other *B. cereus* strains to gain more information of this strain. These results may be useful in elucidating the pathogenicity of *B. cereus* and its role in food poisoning.

## Methods

### Sample collection, strain isolation, and whole genome sequencing

*Bacillus cereus* FORC_013 was isolated from fried eel in South Korea and cultivated in Brain Heart Infusion (BHI; Difco, Detroit, MI, USA) medium. Genomic DNA was isolated and purified using the MoBio UltraClean Microbial DNA Isolation Kit (MoBio, Carlsbad, CA, USA) following the manufacturer’s recommendations. Approximately 5 μg of genomic DNA was cut into 8–12 kb fragments using the Hydroshear system (Digilab, Marlborough, MA, USA). SMRTbell libraries were prepared for each sample using the DNA Template Prep kit 2.0 (3–10 kb) for SMRT sequencing which was carried out with C2 chemistry on a PacBio RS II system (Pacific Bioscience, Menlo Park, CA, USA). The AMPure XP bead purification system (Beckman Coulter, Brea, CA, USA) was used to purify libraries by removing small fragments (<1.5 kb). An Agilent 12,000 DNA kit (Applied Biosystems, Santa Clara, CA, USA) was used to characterize the size distribution of sheared DNA templates. Sequencing primers were annealed to the templates and DNA polymerase enzyme C2 was added following the manufacturer’s instructions. Loading the enzyme template-complexes and libraries onto 75,000 zero-mode waveguides (ZMWs) was conducted using DNA/Polymerase Binding kit P4 (Pacific Bioscience) according to the manufacturer’s instructions. SMRTbell library sequencing using a 120-min sequence capture protocol with PacBio RS II to maximize read length via the DNA sequencing kit Reagent 2.0 (Pacific Bioscience). The summary of sequencing result is contained in Additional file 1.

### Genome assembly and annotation

Whole genome assembly was performed using the SMRT portal system. Sequencing reads from the PacBio RS II system were assembled using the HGAP assembly-3 algorithm with curation of the genome size parameter which was set to 3 Mb using the Compute Minimum Seed Read Length option while other parameters were set to default [12]. Sequencing errors were removed and a polishing assembly process was repeatedly performed to reduce errors, such as indels in the draft assembly using Quiver until none genomic variants were detected. The genome sequences were assembled into contigs using the PacBio RS II system. The orientation and direction of the assembled sequence was defined via Basic Local Alignment Search Tool (BLAST) and Mummer analyses [13]. BioEdit software was used to curate the polished sequence based on alignment results [14]. We used Rapid Annotation of Prokaryotic Genomes (PROKKA) to predict open reading frame (ORF) count and the rRNA and tRNA contents of *B. cereus* FORC_013. [15] The Rapid Annotation using Subsystem Technology (RAST) [16] was used for SEED annotation with the default settings. Cluster of Orthologous Groups (COG) annotation was conducted using WebMGA [17] and DNAPlotter [18] was employed to generate an annotation map.

### Genome accession numbers

To study the comparative genomics of *B. cereus* strains, 29 complete genome sequences were downloaded from the NCBI database (http://www.ncbi.nlm.nih.gov/genome/genomes/157). The accession numbers for these 29 *B. cereus* sequences are CP009318.1 (03BB102), CP009641.1 (03BB108), CP009941.1 (03BB87), CP009596.1 (3a), CP015727.1 (A1), CP001177.1 (AH187), CP001283.1 (AH820), AE017194.1 (ATCC10987), AE016877.1 (ATCC14579), CP009628.1 (ATCC4342), CP001176.1 (B4264), CP001746.1 (CI), CP011153.1 (CMCCP0011), CP011151.1 (CMCCP0021), CP009300.1 (D17), CP009968.1 (E33L), CP003187.1 (F837/76), CP009369.1 (FM1), CP009686.1 (FORC_005), CP012691.1 (FORC_024), CP003747.1 (FRI-35), CP008712.1 (FT9), CP009590.1 (G9241), CP001186.1 (G9842), CP011155.1 (HN001), AP007209.1 (NC7401), CP012483.1 (NJ-W), CP000227.1 (Q1), and CP009605.1 (S2-8).

### Analysis of virulence factors

To investigate the virulence factor encoding genes of FORC_013 strain, we downloaded full DNA sequences from the virulence factor database (VFDB). For virulence gene identification, we used BLASTn method against VFDB (identity ≥0.95).

### Phylogenetic and comparative genome analysis

JSpecies software was employed to compute Average nucleotide identity (ANI) values of all 30 strains [19]. The MESTORTHO algorithm [20] was used to build an orthologous gene set for the 30 complete genomes (identity ≥ 0.95; coverage ≥ 0.8). Multiple sequence alignment of each orthologous gene was conducted using PRANK [21]. After removing the poorly aligned positions using Gblocks [22], orthologous sequences were joined into one sequence to build a phylogenetic tree. The neighbor-joining method was used to construct a tree using MEGA7 [23]. The Codeml program based on PAML4 (phylogenetic analysis by maximum likelihood) [24] was used to detect the genes which were under selective pressure by estimating dN (rate of non-synonymous substitution) and dS (rate of synonymous substitution) based on the branch and branch-site models. Prior to pan-genome analysis, 30 complete genome sequences of *B. cereus* were annotated using the PROKKA annotation tool [15]. After annotation, GFF files output from PROKKA were used as the input files for creating the pan-genome with Roary software [25].

### Quality assurance

The 16s rRNA gene was identified from the assembled sequence using PROKKA. Pairwise distances were calculated by comparison of FORC_013 with published *B. cereus* genomes using ANI values.

## Results and discussion

### Genome features of *Bacillus Cereus* FORC_013

**Fig. 1:**
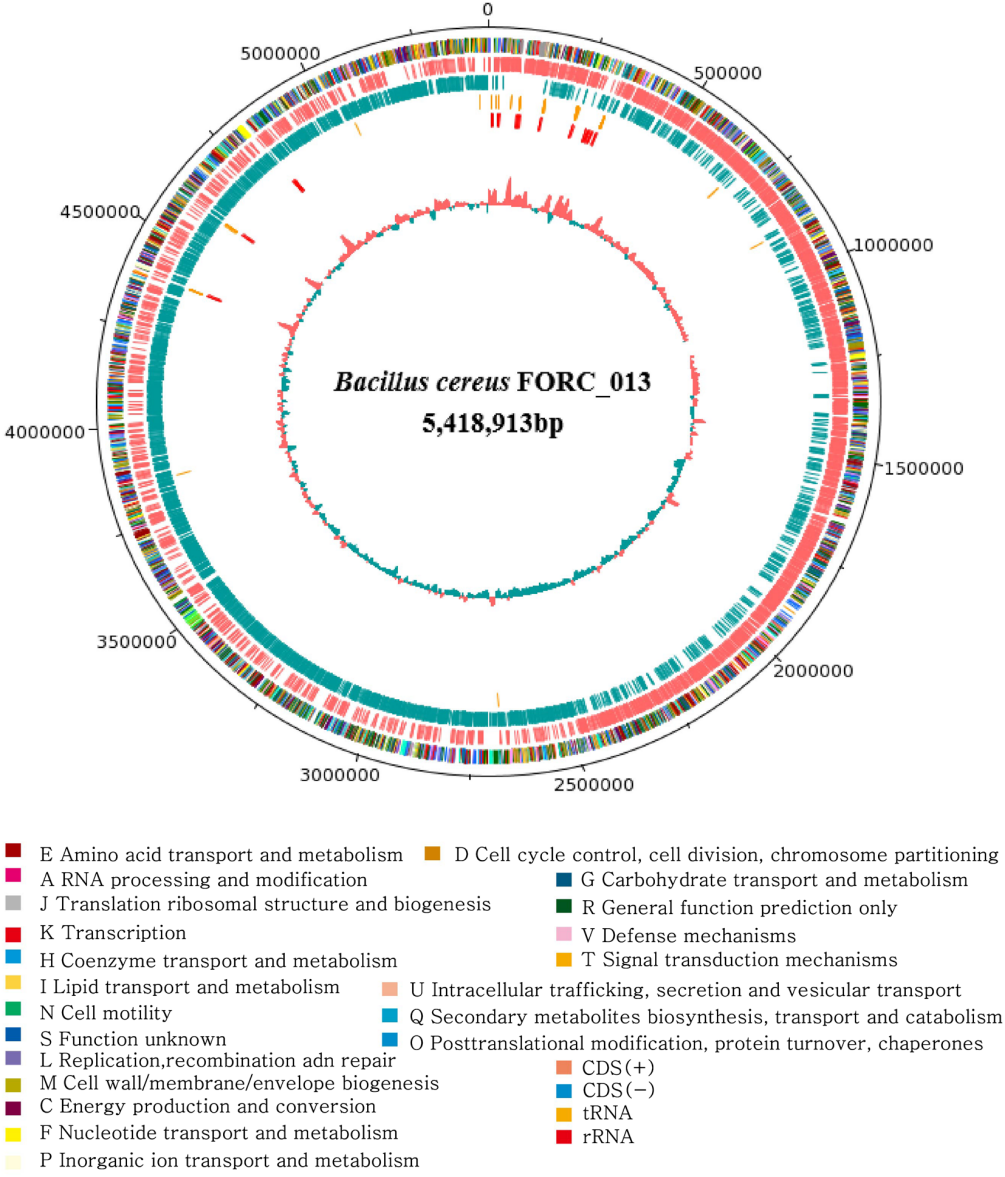
Circular genome map of the *B. cereus* FORC_013 chromosome. *Circles*, from outer to inner, represent the Cluster of Orthologous Groups (COG) distribution, with protein coding sequences (CDS) in the leading strand, CDS in the lagging strand, tRNA, rRNA, and the GC content. Functional genes are labeled around the outer circle as evolutionarily selected genes

**Fig. 2:**
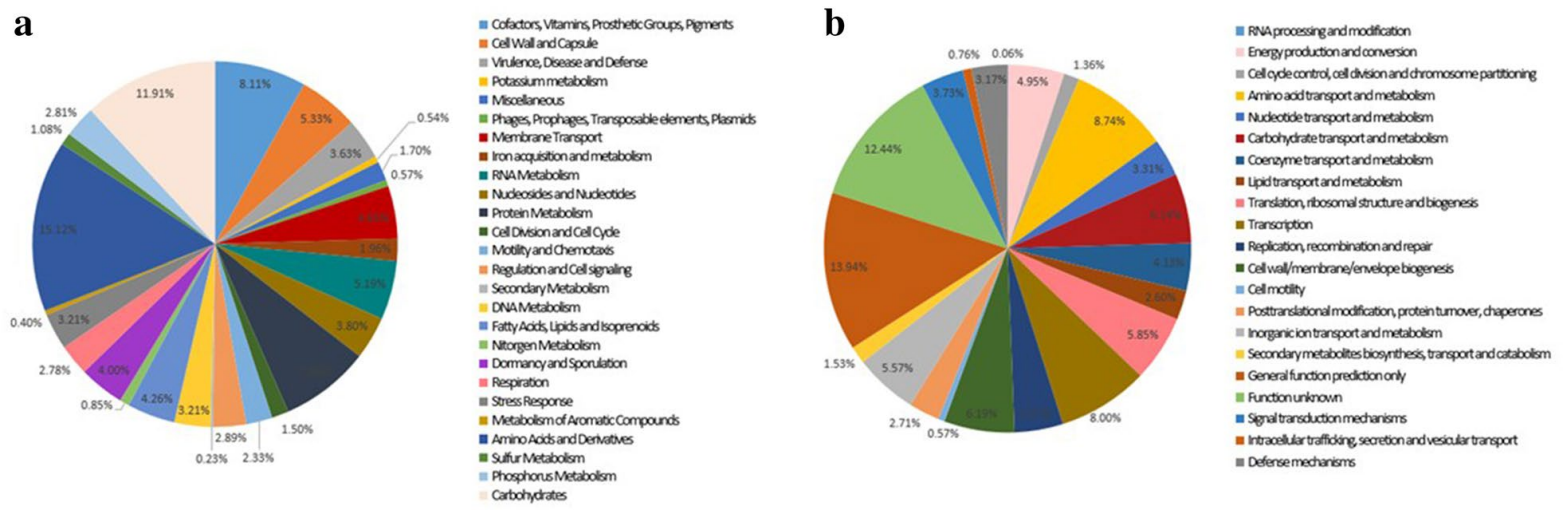
Functional categorization of all estimated open reading frames (ORFs) in the *B. cereus* FORC_013 genome based on the **a** SEED and **b** COG databases

The *B. cereus* FORC_013 genome consists of a circular DNA chromosome and a single circular plasmid (Additional file 1: Table S1). The whole genome sequence comprises 5,418,913 bp with a GC content of 35.3%. The *B. cereus* FORC_013 plasmid contains 259,749 bp with a GC content of 33% and a total of 259 predicted ORFs. The FORC_013 genome contains 5424 ORFs, 107 tRNA sequences and 42 rRNA sequences. Among the predicted ORFs, 3750 (69%) were predicted based on annotated genes and 1674 (31%) were hypothetical and unknown proteins (Fig. 1). Figure 2 presents the categorization of estimated functional genes based on SEED subsystem categories and COG functional categories; 3525 genes were classified into 26 SEED subsystem categories. Of these, 286 ORFs were categorized into the cell wall and capsule subsystem, which includes pathogenicity; 128 ORFs were responsible for virulence, disease and defense, which may be related to toxin production. In total, 82 ORFs were related to motility and chemotaxis due to the formation of biofilms, which affect the persistence of the pathogen. Functional annotation based on COG categorization using WebMGA identified 3537 ORFs. Excluding ORFs that were related to the general function prediction only and function unknown categories (26.4%), 1235 ORFs, accounting for more than one-third of the COG-assigned ORFs, were classified into five major COG categories: 309 ORFs in category E (amino acid transport and metabolism), 283 ORFs in category K (transcription), 219 ORFs in category M (cell wall/membrane/envelope biogenesis), 217 ORFs in category G (carbohydrate transport and metabolism) and 207 ORFs in category J (translation, ribosomal structure and biogenesis).

### Virulence factors

The 20 virulence genes of FORC_013 were identified via BLASTn method against VFDB (Table 1). The virulence factors of FORC_013 were classified into six categories: host immune evasion, lipase, protease, regulation, toxin, and others. As previous studies reported, the diarrheal symptom is well known for having a close relationship with the enterotoxin, such as hemolytic enterotoxin HBL, non-hemolytic enterotoxin NHE and cytotoxin K [7, 11]. The genome of FORC_013 has all of these enterotoxins; *Cyt*K gene, HBL gene cluster (*hbl*A, *hbl*B, *hbl*C, and *hbl*D) and NHE gene cluster (*nhe*A, *nhe*B, and *nhe*C), suggesting that these genes are responsible for pathogenicity of FORC_013. In the protease category, immune inhibitor A metalloprotease (*inh*A) was detected; this gene assists in surviving the macrophage environment, which is an important factor of the host immune system [26]. Further, this supports that *inh*A in FORC_013 may contribute to retain living in the macrophage intracellular system. The FORC_013 strain has hemolysin II (*hyl* II) and hemolysin III (*hyl* III) that form the pores by adapting under the harsh environment [27, 28]. We also identified an regulation protein, pleiotropic regulator (*Plc*R), which is a well-known pleiotropic regulator of genes related to pathogenicity [29]. This gene plays a role in the biofilm formation, which may induce the sporulation of bacteria [30, 31]. Biofilm formation facilitates generating adhesive spores and contributes to high resistance [32]. Detection of *Plc*R indicated that the FORC_013 may take advantage of both biofilm formation and virulence gene regulation. Based on the results, it is reasonable to assume that these virulence factors contribute to pathogenicity of FORC_013. Additionally, we conducted a lactate dehydrogenase (LDH) release assay to identify cytotoxicity, which indicated that FORC_013 has pathogenic activity (Additional file 1: Fig S1).

**Table 1:** Virulence factors of *B. cereus* FORC_013

Virulence factor	Annotation	Locus tag
Host immune evasion		
–	Polysaccharide capsule	FORC13_5198, FORC13_5217
Lipase		
*plcA*	Phosphatidylcholine-preferring phospholipase C (PC-PLC)	FORC13_4514
*piplc*	Phosphatidylinositol-specific phospholipase C (PI-PLC)	FORC13_1400
Protease		
*inhA*	Immune inhibitor A metalloprotease	FORC13_4518
–	Immune inhibitor A metalloprotease	FORC13_3892
Regulation		
*plcR*	PlcR	FORC13_5291
Toxin		
–	Anthrolysin O	FORC13_5042
*cytK*	Cytotoxin K	FORC13_4071
*hlyII*	Hemolysin II	FORC13_1637
*hlyIII*	Hemolysin III	FORC13_3034
–	Hemolysin III homolog	FORC13_5388
*hblC, hblD, hblB, hblA*	Hemolytic enterotoxin HBL	FORC13_2078~FORC13_2081
*nheC, nheB, nheA*	Non-hemolytic enterotoxin NHE	FORC13_3362~FORC13_3364
Others		
–	Internalin-like	FORC13_4633
–		FORC13_3846

### Phylogenetic and comparative genome analysis

**Fig. 3:**
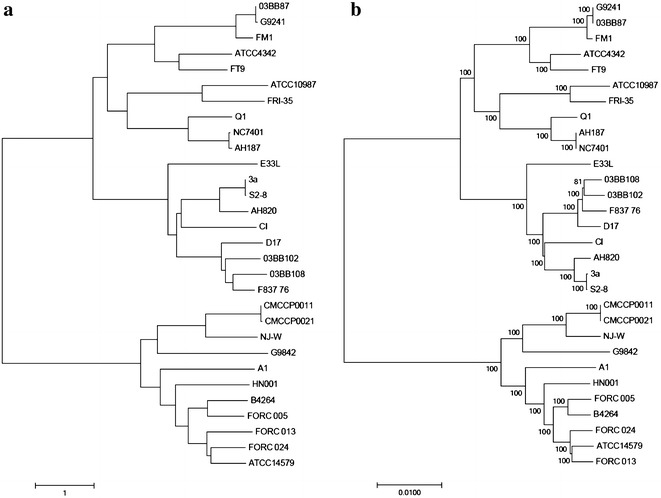
Average nucleotide identity (ANI) tree and phylogenetic tree based on **a** ANI value and **b** orthologous genes, respectively

An ANI tree and a phylogenetic dendrogram based on orthologous genes were built for a comparative analysis of the FORC_013 strain. Both trees were generated using 29 complete genome sequences acquired from the NCBI database and the FORC_013 genome sequence (Fig. 3). The neighbor-joining method was used to construct an ANI tree with pairwise distance matrix and a phylogenetic tree with orthologous genes. The ATCC14579 strain was shown to contain pathogenicity-related genes in a previous study [33]. In both of our tree analyses, FORC_013 clustered closely with ATCC14579. The high ANI value (98.6%) indicates that FORC_013 may have genes affecting virulence, similar to ATCC14579.

To identify the positively selected genes, we calculated the positive selection sites for the orthologous gene using the branch and branch-site models. In the branch model, 12 genes were revealed as being selected: YtxC-like family protein, post-transcriptional regulator ComN, cytochrome c-550, HTH-type transcriptional regulator NorG, putative HTH-type transcriptional regulator, flagellar hook-basal body complex protein FliE, putative murein peptide carboxypeptidase, HTH-type transcriptional regulator GltC, AT synthase subunit a, regulatory protein YeiL, superoxide dismutase (Mn)2, and oligoendopeptidase F (Additional file 1: Table S2). In the branch-site model, two putative genes were detected with their own functions: mycinamicin III 3′′-*O*-methyltransferase and putative efflux system component YknX (Additional file 1: Table S3). In the methyltransferase gene, amino acid 190 was changed to asparagine from aspartic acid in FORC_013. Asparagine and aspartic acid are categorized in the carboxamide group and the negatively charged group, respectively. In the gene identified as a putative efflux system component, amino acid 153 in FORC_013 was changed to isoleucine from valine. Isoleucine is in the hydrophobic group, while valine is categorized as non-polar. Through evolutionary analysis, we detected some positively selected genes related to virulence. The superoxide dismutase (Mn) 2 plays a crucial role in protecting cells from the oxidative stress [34, 35]. Toxin from FORC_013 can survive low gastric pH condition in the presence of the superoxide dismutase (Mn) 2. The YknX gene encodes an ABC transporter, which is contributed to the export of virulence factor [36, 37]. These results suggest that the positively selected genes identified in the FORC_013 strain may have an influence on pathogenicity.

Furthermore, Pan-genome analysis of 30 strains revealed 25,247 genes comprising the supra-genome based on the Roary pipeline (Additional file 1: Fig S2a). The relation between the number of genomes (*x*) and the pan-genome size (*y*) was *y* = 7520.62*x* ^0.37^ − 2066.4 (*R* ^*2*^ = 0.999926). Also, the relationship between the core genome size and the genome number was calculated as *n* = 7276.95e^−0.82*m*^ + 2284.85 *(R* ^*2*^ = 0.960822). The size of the *B. cereus* pan-genome has grown, while the scale of core genome has decreased with the addition of new strains (Additional file 1: Fig S2b). Based on this result, we can consider this pan-genome to be an open pan-genome, providing evidence that this species dwells under conditions that encourage the transfer of genetic material through pathways such as horizontal gene transfer [38, 39]. Above all, we examined the unique genes of FORC_013 to elucidate the strain’s specific biological characteristics. We detected that the unique genes of the FORC_013 strain comprise 224 genes, including 130 hypothetical proteins (Additional file 2: Table S1). The proportion of unique genes of FORC_013 was 4.16% (Additional file 1: Fig S2c). Furthermore, we could detect strain-specific genes of FORC_013 associated with virulence through Pan-genome analysis. Here, we identified A-type flagellin and flagellin genes that are involved in biofilm formation [40], which are candidates for assisting the activation of FORC_013’s pathogenicity.

## Conclusions

In this study, we sequenced the genome of *B. cereus* FORC_013, which is an opportunistic pathogen that occurs food-borne illness, and performed comparative analysis with 29 published strains. As a result, we detected the virulence factors of this strain that can assist its pathogenicity. We also identified positively selected genes and unique genes of FORC_013. This study advances our understanding of the genetic characteristics of FORC_013. In addition, these findings will provide useful information for further research related to the virulence mechanisms used by this pathogen.

## Additional files

**Additional file 1.** Additional Tables and Figures.

**Additional file 2: Table S1.** Additional Tables.

## Abbreviations

ORFs: open reading frames
PROKKA: rapid annotation of prokaryotic genomes
RAST: rapid annotation using subsystem technology
COG: Cluster of Orthologous Groups
BLAST: basic local alignment search tool
ANI: average nucleotide identity
CDS: protein coding sequences
