# Supplementary material for: Comparative genomic analysis reveals genetic features related to the virulence of Bacillus cereus FORC_013
Source: Gut Pathog. 2017 May 15;9:29. doi: 10.1186/s13099-017-0175-z (PMC5433235; doi:10.1186/s13099-017-0175-z)

**Additional Files**

**Comparative genomic analysis reveals genetic features related to the virulence of *Bacillus cereus* FORC_013**

Hyun-Jin Koo^1^, Sojin Ahn^2,3^, Han Young Chung^3,4^, Suyeon Kim^3,4^, Kwondo Kim^2^, Sangryeol Ryu^3,4^, Ju-Hoon Lee^3,5^, Sang Ho Choi^3,4*^ and Heebal Kim^1,2,3*^

**Author details**

^1^Department of Agricultural Biotechnology and Research Institute of Agriculture and Life Sciences, Seoul National University, Seoul, Republic of Korea

^2^Interdisciplinary Program in Bioinformatics, Seoul National University, Seoul, Republic of Korea

^3^Food-borne Pathogen Omics Research Center (FORC), Seoul National University, Seoul, Korea

^4^Department of Agricultural Biotechnology, Center for Food Safety and Toxicology, Seoul National University, Seoul, Republic of Korea

^5^Department of Food Science and Biotechnology, Institute of Life Science and Resources, Kyung Hee University, Yongin, Republic of Korea

***Correspondence:**

[heebal@snu.ac.kr](mailto:heebal@snu.ac.kr); choish@snu.ac.kr

^1^Department of Agricultural Biotechnology and Research Institute of Agriculture and Life Sciences, Seoul National University, Seoul, Republic of Korea

^4^Department of Agricultural Biotechnology, Center for Food Safety and Toxicology, Seoul National University, Seoul, Republic of Korea

**Material and Methods**

**Cytotoxicity test**

Cytotoxicity test of FORC_013 strain was performed via checking cytoplasmic lactated dehydrogenase (LDH) activity which is observed from the human epithelial INT-407 cells that were lysed by 2% Triton X 100. As mentioned previously, the INT-407 cells were mature in minimum essential medium containing 1% (v/v) fetal bovine serum (MEMF) (Gibco-BRL, Gaithersburg, MD) in 96-well culture dishes (Nunc, Roskilde, Denmark) [[1](#_ENREF_1)]. Each well of the INT-407 2 × 10^4^ cells was infected with *B. cereus* FORC_013 and ATCC 14579 as positive control at various multiplicities of infections (MOIs) for 3hours. The LDH releasing activity into the supernatant was determined via a cytotoxicity detection kit (Roche, Mannheim, Germany).

**References**

1. Kim S, Bang YJ, Kim D, Lim JG, Oh MH, Choi SH: **Distinct characteristics of OxyR2, a new OxyR‐type regulator, ensuring expression of Peroxiredoxin 2 detoxifying low levels of hydrogen peroxide in Vibrio vulnificus**. *Molecular microbiology* 2014, **93**(5):992-1009.

**Additional file 1: Table S1. Summary of *B. cereus* FORC_013 genome.**

| **Property** | **Term** |
| --- | --- |
| Finishing quality | Finished |
| Libraries used | PacBio SMRTbell™ library |
| Number of SMRT cells | 2 |
| Sequencing platforms | PacBio RS II sequencer |
| Assemblers | PacBio SMRT analysis 3.0 |
| Gene calling method | PROKKA, and RAST |
| Number of reads | 109,880 (PacBio_20K) |
| N50 read length | 7,373 |
| Average genome coverage | 71.39x |
| Contigs no. | 2 (chromosome) |
|  | 1 (plasmid) |
| Scaffolds no. | 1 (chromosome) |
|  | 1 (plasmid) |
| N50 contig length | 2,948,960 |
| Chromosome length (bp) | 5,418,913 bps (chromosome)  259,749 bps (plasmid) |
| ORFs | 5,424 |
| Locus Tag | FORC13 |
| Genbank ID | CP011145, CP011146 |
| BIOPROJECT | PRJNA279901 |
| Source Material Identifier | FORC_013 |

**Additional file 1: Fig S1. Cytotoxicity analysis for two strains of *B. cereus*** The cytotoxicity analyses of FORC_013 strain were compared with ATCC 14579 strain by measuring the activity of cytoplasmic lactate dehydrogenase (LDH). INT-407 cells were infected with FORC_013 or ATCC 14579 at various multiplicities of infection (MOIs) for 3 h. Cytotoxicity was determined as the percentage of LDH leakage using the amount of LDH from the cells that were completely lysed by 2% Triton X-100. Error bars represent the standard errors of the means (SEM).


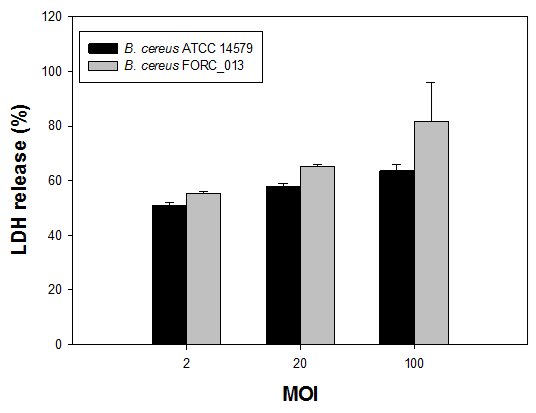


**Additional file 1: Table S2. Positively selected genes predicted in the branch model and related data for *B. cereus* FORC_013**

| **Locus** | **Function** | **p-value** | **ω_F.G._** | **ω_B.G._** |
| --- | --- | --- | --- | --- |
| FORC13_0586 | YtxC-like family protein | 2.04E-02 | 1.03927 | 0.04993 |
| FORC13_0734 | Post-transcriptional regulator ComN | 4.62E-02 | 1.0206 | 0.05055 |
| FORC13_0851 | Cytochrome c550 | 2.09E-05 | 1.00565 | 0.07063 |
| FORC13_1405 | HTH-type transcriptional regulator NorG | 3.91E-11 | 1.09935 | 0.05774 |
| FORC13_3326 | Putative HTH-type transcriptional regulator | 5.13E-03 | 1.11237 | 0.03878 |
| FORC13_3534 | Flagellar hook-basal body complex protein FliE | 2.93E-02 | 1.0579 | 0.10196 |
| FORC13_3811 | putative murein peptide carboxypeptidase | 7.82E-03 | 1.21275 | 0.11958 |
| FORC13_5156 | HTH-type transcriptional regulator GltC | 4.76E-21 | 1.18227 | 0.0696 |
| FORC13_5251 | ATP synthase subunit a | 1.41E-02 | 1.05517 | 0.12087 |
| FORC13_5350 | Regulatory protein YeiL | 2.27E-04 | 2.89351 | 0.13594 |
| FORC13_5384 | Superoxide dismutase (Mn) 2 | 3.19E-03 | 1.18661 | 0.04302 |
| FORC13_5392 | Oligoendopeptidase F | 1.52E-03 | 1.28818 | 0.07277 |

**Additional file 1: Table S3. Positively selected genes predicted in the branch-site model and related data for *B. cereus* FORC_013**

| **Function** | Mycinamicin III 3''-O-methyltransferase | Putative efflux system component YknX |
| --- | --- | --- |
| **Locus position** | FORC13_3977 | FORC13_5192 |
| **Peptide length** | 258 | 395 |
| **p-Value** | 8.77E-20 | 2.04E-02 |
| **ω2 F.G.** | 999.00 | 74.42 |
| **ω2a B.G.** | 0.0009 | 0.0185 |
| **Proportion of site class 2a** | 0.893 | 0.934 |
| **Bayes Empirical Bayes** | 0.967* | 0.975* |
| **Position** | 190 | 153 |
| **FORC_013** | N | I |
| **Other strains** | D | V |

**Additional file 1: Fig S2. Pan-genome- each strain is represented by a vertical line** (a) pan-genome structure for 30 *B. cereus* species (b) core genome and pan genome (c) proportion of core, dispensable, and unique genes.


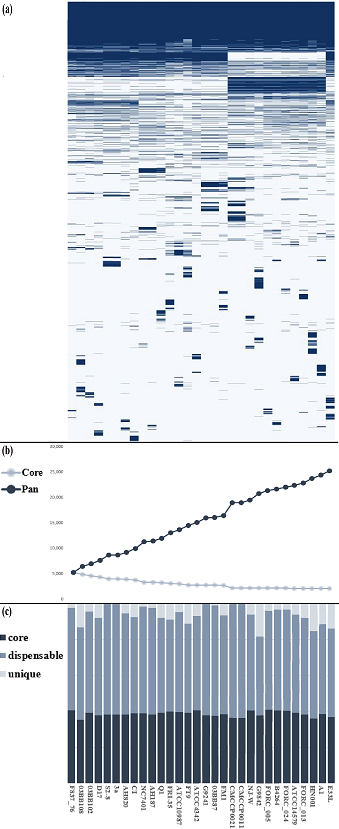

Supplement: Supplementary file 1 — Additional file 1. Additional Tables and Figures. [file 13099_2017_175_MOESM1_ESM.docx]
